# Dietary Supplementation of Blueberry Juice Enhances Hepatic Expression of Metallothionein and Attenuates Liver Fibrosis in Rats

**DOI:** 10.1371/journal.pone.0058659

**Published:** 2013-03-12

**Authors:** Yuping Wang, Mingliang Cheng, Baofang Zhang, Fei Nie, Hongmei Jiang

**Affiliations:** 1 Department of Clinical Microbiology and Immunology, Affiliated Hospital of Guiyang Medical College, Guiyang, Guizhou Province, China; 2 Department of Infectious Diseases, Affiliated Hospital of Guiyang Medical College, Guiyang, Guizhou Province, China; 3 Guizhou Academy of Sciences, Guiyang, Guizhou Province, China; National Institutes of Health, United States of America

## Abstract

**Aim:**

To investigate the effect of blueberry juice intake on rat liver fibrosis and its influence on hepatic antioxidant defense.

**Methods:**

Rabbiteye blueberry was used to prepare fresh juice to feed rats by daily gastric gavage. Dan-shao-hua-xian capsule (DSHX) was used as a positive control for liver fibrosis protection. Liver fibrosis was induced in male Sprague-Dawley rats by subcutaneous injection of CCl_4_ and feeding a high-lipid/low-protein diet for 8 weeks. Hepatic fibrosis was evaluated by Masson staining. The expression of α-smooth muscle actin (α-SMA) and collagen III (Col III) were determined by immunohistochemical techniques. The activities of superoxide dismutase (SOD) and malondialdehyde (MDA) in liver homogenates were determined. Metallothionein (MT) expression was detected by real-time RT-PCR and immunohistochemical techniques.

**Results:**

Blueberry juice consumption significantly attenuates CCl_4_-induced rat hepatic fibrosis, which was associated with elevated expression of metallothionein (MT), increased SOD activity, reduced oxidative stress, and decreased levels of α-SMA and Col III in the liver.

**Conclusion:**

Our study suggests that dietary supplementation of blueberry juice can augment antioxidative capability of the liver presumably via stimulating MT expression and SOD activity, which in turn promotes HSC inactivation and thus decreases extracellular matrix collagen accumulation in the liver, and thereby alleviating hepatic fibrosis.

## Introduction

Hepatic fibrosis is a repair response to chronic liver injury [1]. It is a progressive pathological process and a common pathological change in chronic liver diseases. Oxidative stress is an important pathogenic factor for many liver diseases, which can cause hepatocyte damage through lipid peroxidation and protein alkylation [2]–[5]. Superoxide dismutase (SOD) and catalase are important antioxidant enzymes that function as endogenous free radical scavengers. Recently, metallothionein (MT) was identified as a more efficient scavenger for reactive oxygen species (ROS) [6]. There are two major isoforms of MT, Mt-1 and Mt-2, which are ubiquitously distributed in almost all tissues [7]. Interestingly, MT was shown to be able to enhance SOD activity in vitro [8]. Cardiac overexpression of MT has been shown to effectively attenuate diabetic cardiomyopathy via suppression of reactive oxygen species (ROS) production and oxidative stress [9]. Currently, there are no effective drugs available to prevent or treat liver fibrosis, and some natural substances with antioxidant properties are under investigation for developing new therapeutic reagents.

Blueberries are perennial flowering plants belonging to *Vaccinium spp.* of the family *Ericaceae.* In recent years, Human Nutrition Research Center of the United States has carried out a series of studies, demonstrating that blueberry contains a high level of anthocyanins and appears to have the highest antioxidant capacity among fruits and vegetables [10], [11]. Blueberry and probiotics were shown to have protective effects on acute liver injury induced by d-galactosamine and lipopolysaccharide [12]. Proanthocyanidin isolated from blueberry leaves was able to inhibit hepatitis-c virus (HCV) replication [13]. Furthermore, proanthocyanidin derived from the leaves of *Vaccinium virgatum* suppresses platelet-derived growth factor-induced proliferation of the human hepatic stellate cell line LI90 [14]. Increasing blueberry consumption seems to be a practical and effective strategy to reduce oxidative stress and thus protect tissue injury [15].

The aim of this research work was to examine the effect of blueberry juice on CCl_4_-induced rat liver fibrosis and its effects on hepatic levels of the endogenous antioxidant components.

## Materials and Methods

### Reagents and animal treatments

“Rabbiteye Blueberry” was produced by Guizhou Academy of Sciences in the Blueberry Production Field at Ma-Jiang, Guizhou, China. The blueberry was stored at −20°C until experimental use. Fresh blueberry juice was prepared by homogenization of the frozen fruit right before the start of experiments (1 ml of blueberry juice contained about 2 g of dried blueberry). The major active ingredients of the blueberry juice was analyzed by HPLC-DAD and NBT methods and shown in Table 1. Dan-shao-hua-xian capsule (DSHX) capsules composed of five Chinese herbal medicines-Tetrandrine, Radix Salviae Miltiorrhizae, Radix Paeoniae Rubra, Astragalus Membranaceus and Ginkgleaf was purchased from Guiyang Pharmaceutical Company (Guizhou, China, lot number 20081011). We previously reported that DSHX is effective in preventing hepatic fibrosis [16], and it was therefore served as positive control for liver fibrosis protection.

**Table 1:** Major active ingredients of the blueberry juice.

Ingredients	Anthocyanin(mg/kg)	SOD (U/kg)
	3507.6±56.12	3.7×10^4^±101.23

Forty-five male Sprague–Dawley rats (200±20 g) were obtained from the Experimental Animal Center of Guiyang Medical College, Guiyang, China (Approval number SCXK (Guizhou) 2002-0001). Rats were randomly divided into five groups with nine rats in each group: control group, CCl_4_-induced hepatic fibrosis group (model group), blueberry juice prevention group (BB group), Dan-shao-hua-xian capsule prevention group (DSHX group), and blueberry juice+DSHX prevention group (BB+DSHX group). Except in the control group, liver fibrosis was induced in by a complex method [17]. Rats in model group, BB group, DSHX group, and BB+DSHX group received subcutaneous injections of 40% CCl_4_ solution (mixture of pure CCl_4_ and peanut oil) at a dose of 3 ml/kg twice a week for 8 weeks (the first dose was 4 ml/kg). Rats were fed a high-lipid/low-protein diet (79.5% corn farina, 20% fat, and 0.5% cholesterol) each day. Rats in control group were fed a normal diet. At the same time, blueberry juice (15 g/kg, once a day, by gastric gavage), DSHX (1.0 g/kg, once a day, by gastric gavage), and blueberry juice+DSHX (15 g/kg, and 1.0 g/kg, once a day, by gastric gavage) were given to the rats in the corresponding groups. After 8 weeks, rats were killed to collect blood and livers. The same part of each liver was removed and fixed in 10% neutral formalin; the remaining portion of liver was stored at −80°C. Rat serum was prepared by centrifugation (1500 rpm for 15 min at room temperature) and stored at −80°C.

All animal studies complied with the Animal Care and Use Guidelines of Guiyang Medical College (Guiyang, China).

### Histopathology and the measurement of serum AST levels

After fixation in 10% formalin for 24 h, liver samples were embedded in paraffin. Samples were then cut into 5-µm pieces and mounted on slides. They were then stained with hematoxylin and eosin (H&E) for histopathological examination, and with fibrosis-specific Masson stain for evaluation of the degree of liver fibrosis. Histologic evaluation was performed twice by two pathologists blinded to the protocol on five low-power fields per slide. Modified Ishak scoring system was used with minor changes in order to define the degree of fibrosis [18].

Concentrations of serum AST were measured by an automatic biochemical analytic instrument (Siemens Advia 1650, Bensheim, Germany).

### Immunohistochemical analysis

After de-paraffinization, rehydration, and antigen unmasking by heat treatment, liver sections were incubated in 3% H_2_O_2_ for 10 min. They were then incubated with anti-α-SMA (SC-32251, Santa Cruz Biotechnology, Santa Cruz, CA, USA), anti-Col III (SC-80564, Santa Cruz Biotechnology) and anti-MT (sc-11377, Santa Cruz Biotechnology) antibody (1∶100) overnight at 4°C. Samples were then processed using an EnVision kit (lot number 10N1775A, Dako, Denmark) according to the manufacturer's protocols. Phosphate-buffered saline (PBS) was used as negative control. The cells with brown staining in the cytoplasm/nucleus were considered to be positive. Five high power microscopic fields (400× magnification) were randomly chosen per slide, and the number of positive cells per field was counted (α-SMA and MT immunopositivity). For Col III staining, 5 high power fields were chosen randomly in each section and the images were analyzed by the Biomias2000 Image Analytic Instrument to quantify the transparency of the immunopositive areas in the section (data expressed as optical density (OD) that is in inverse proportion to the intensity of immunostaining signals).

### Real-time reverse transcriptase-polymerase chain reaction (RT-PCR) analysis

Total RNA was extracted from liver tissues with Trizol reagent (lot number 13827390, Invitrogen, Carlsbad, CA, USA) according to the manufacturer's instructions. The RNA samples were used for reverse transcription with Moloney murine leukemia virus (MMuLV) reverse transcriptase and oligo (dT) primers (lot number 00033699, Fermentas, MBI, Burlington, ON, Canada). The SYBR Green DNA PCR kit (lot number 0804104, Applied Biosystems, Foster City, CA, USA) was used for real-time RT-PCR analysis. PCR primers were supplied by Dr. Jie Liu (The University of Kansas Medical Center, USA). The sequences of the primers are as follows. MT-I upstream 5′-TGTGCCTGAAGTGACGAACAG-3′,downstream 5′-TTCACATGCTCGGTAGAAAACG-3′; Beta-actin upstream 5′-TCCTCCTGAGCGCAAGTACTCT-3′,downstream 5′-GCTCAGTAACAGTCCGCCTAGAA-3′.

The cycle time (C_t_) values of the genes of interest were normalized with β-actin from the same sample. Relative differences between groups were calculated and expressed as relative increases, with the control set to 100%.

### Measurement of levels of SOD and MDA in liver homogenates

Liver homogenates were prepared using the frozen liver tissues. SOD content was determined by the xanthine oxidase method and MDA content was tested by the thiobarbituric acid method according to the manufacturer's instructions (lot numbers 20090615, 20090616, respectively; Jiancheng Biologic Company, Nanjing, China).

### Statistical analysis

Data analysis was carried out using SPSS 11.5 software (Chicago, IL, USA). Quantitative data were expressed as mean±SD and subjected to one-way analysis of variance, followed by Tukey's Post Test for multiple comparisons. Ordinal data were analyzed by Radit analysis. *P*<0.05 was considered statistically significant.

## Results

### Blueberry juice consumption attenuates CCl_4_-induced rat hepatic fibrosis and liver injury

**Figure 1:**
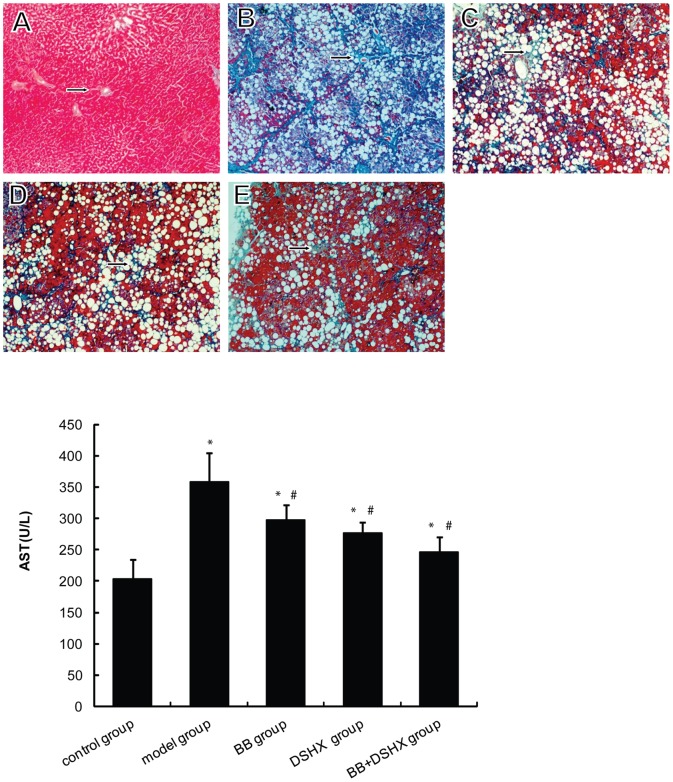
Effect of blueberry juice on CCl_4_-induced liver fibrosis and liver damage. Liver fibrosis was induced in rats by subcutaneous injection of CCl_4_ and subsequently feeding a high-lipid/low-protein diet for 8 weeks with or without daily blueberry juice gavage. After 8 weeks, the degree of hepatic fibrosis was evaluated by Masson staining. ***The upper panel images*** show the representative liver histology changes in the livers of the normal control group (**A**), CCl_4_-induced hepatic fibrosis group (the model group, **B**), blueberry juice prevention group (BB group, **C**), Dan-shao-hua-xian capsule prevention group (DSHX group, **D**), and blueberry juice+DSHX prevention group (BB+DSHX group, **E**), Original magnification 100×, arrows indicate collagen fibers. ***The lower panel*** illustrates serum aspartate aminotransferase (AST) levels in the five groups of rats as indicated. *p<0.05 *vs.* the control group, #p<0.05 *vs.* the model group.

We established a rat hepatic fibrosis model with chronic CCl_4_ injection and the degree of liver fibrosis was assessed by H&E and Masson staining (Figure 1). In control group, hepatocytes had a normal radial array surrounding the central veins, and no regenerating collagen fibers were present. In the fibrosis model group, the lobular structure of hepatocytes was destroyed, and the hepatic plates were disordered with diffuse, fatty degeneration. Collagen fibers expanded into the hepatic parenchyma, which formed fibrous septa surrounding and separating the normal lobules. Pseudolobules were observed in a few samples, and infiltration of numerous inflammatory cells was present in the portal area and fibrous septa. These histopathological changes indicate significant liver fibrotic changes in rats chronically treated with CCl_4_. The hepatic fibrosis was significantly alleviated in BB group, DSHX group, and BB+DSHX group, as evidenced by much thinner fibrous septa these groups (Figure 1) with decreased hepatocyte fatty degeneration, bridging fibrosis, and less inflammatory cell infiltration [19]. We also measured serum AST concentrations to determine the degree of liver injury in the rats. Consistent with our histological data, rats fed blueberry juice had significantly lower levels of serum AST compared to the fibrosis model control group (Figure 1).

### Blueberry juice consumption reduces α-SMA and Col III expression in the liver

**Figure 2:**
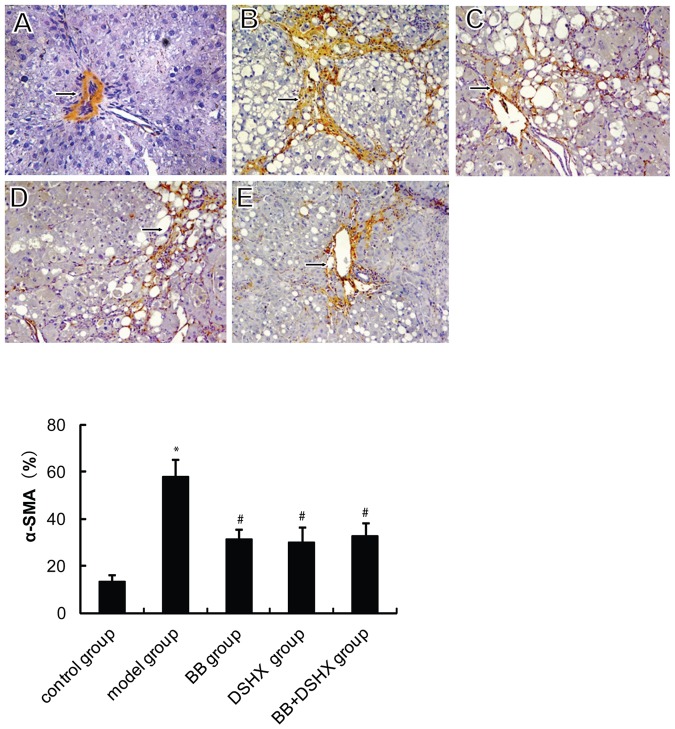
Effect of blueberry juice on α-SMA protein expression in rat livers. The same groups of rats were treated as described in Figure 1. ***The upper panel images ***show the representative liver α-SMA IHC staining in the normal control group (**A**), CCl_4_-induced hepatic fibrosis group (the model group, **B**), blueberry juice prevention group (BB group, **C**), Dan-shao-hua-xian capsule prevention group (DSHX group, **D**), and blueberry juice+DSHX prevention group (BB +DSHXgroup, **E**). Original magnification 400×, arrows indicate positive cytoplasmic staining. ***The lower panel*** shows the quantification data of α-SMA IHC staining. *p<0.05 *vs.* the control group, #p<0.05 *vs.* the model group.

We next examined the effect of blueberry on hepatic α-SMA and Col III expression, two markers for liver fibrosis and hepatic stellate cells (HSCs) activation. Immunohistochemical staining showed that, in control group, α-SMA -positive staining was restricted to the vascular walls in the portal areas and the central veins, while a significantly stronger immune staining was noted in the periportal sinusoid and around the bile ductules in the model group (Figure 2). The expression of α-SMA were significantly decreased in BB group, DSHX group and BB+DSHX group compared with model group (*P*<0.05, Figure 2).

**Figure 3:**
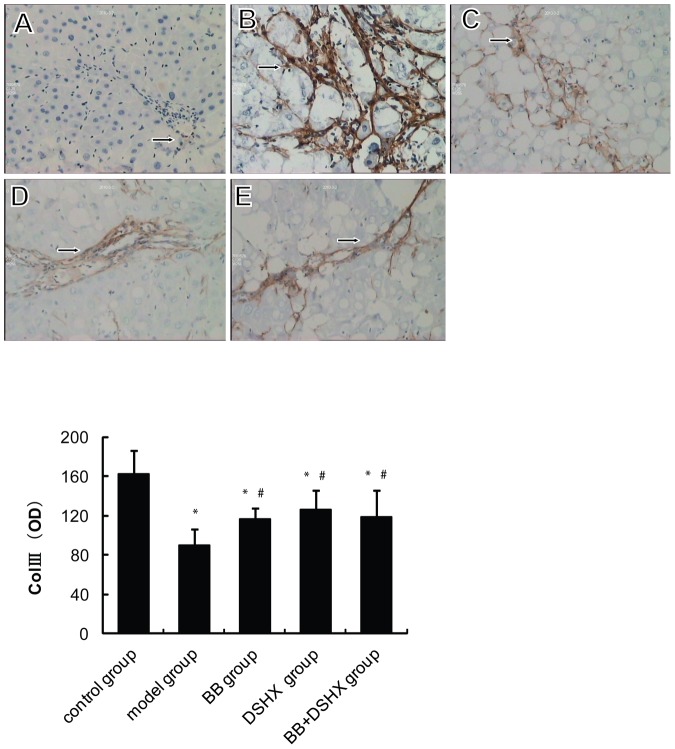
Effect of blueberry juice on Col III protein expression in rat livers. ***The upper panel images*** show the representative liver Col III IHC staining in the normal control group (**A**), CCl_4_-induced hepatic fibrosis group (the model group, **B**), blueberry juice prevention group (BB group, **C**), Dan-shao-hua-xian capsule prevention group (DSHX group, **D**), and blueberry juice+DSHX prevention group (BB+DSHX group, **E**). Original magnification 400×, arrows indicate positive cytoplasmic staining. ***The lower panel ***shows the quantification data of Col III IHC staining by using the Biomias2000 image analysis system, which measures the transparence of the slides that is reversely related to the positivity of Col III IHC staining. *p<0.05 *vs.* the control group, #p<0.05 *vs.* the model group.

A very low level of Col III expression was observed in the normal control group, which was mainly distributed in the periportal areas (Figure 3). In the fibrosis model group, abundant Col III positive fibers were detected in periportal areas and hepatic sinusoids, which expanded into the liver parenchyma to form fibrous septa that surrounded hepatocytes and formed pseudolobules (Figure 3). In the BB group, DSHX group and BB+DSHX group, the expression of Col III was significantly ameliorated compared with the model group (*P*<0.05, Figure 3).

### Effect of blueberry juice on SOD and MDA levels in the rat liver

**Figure 4:**
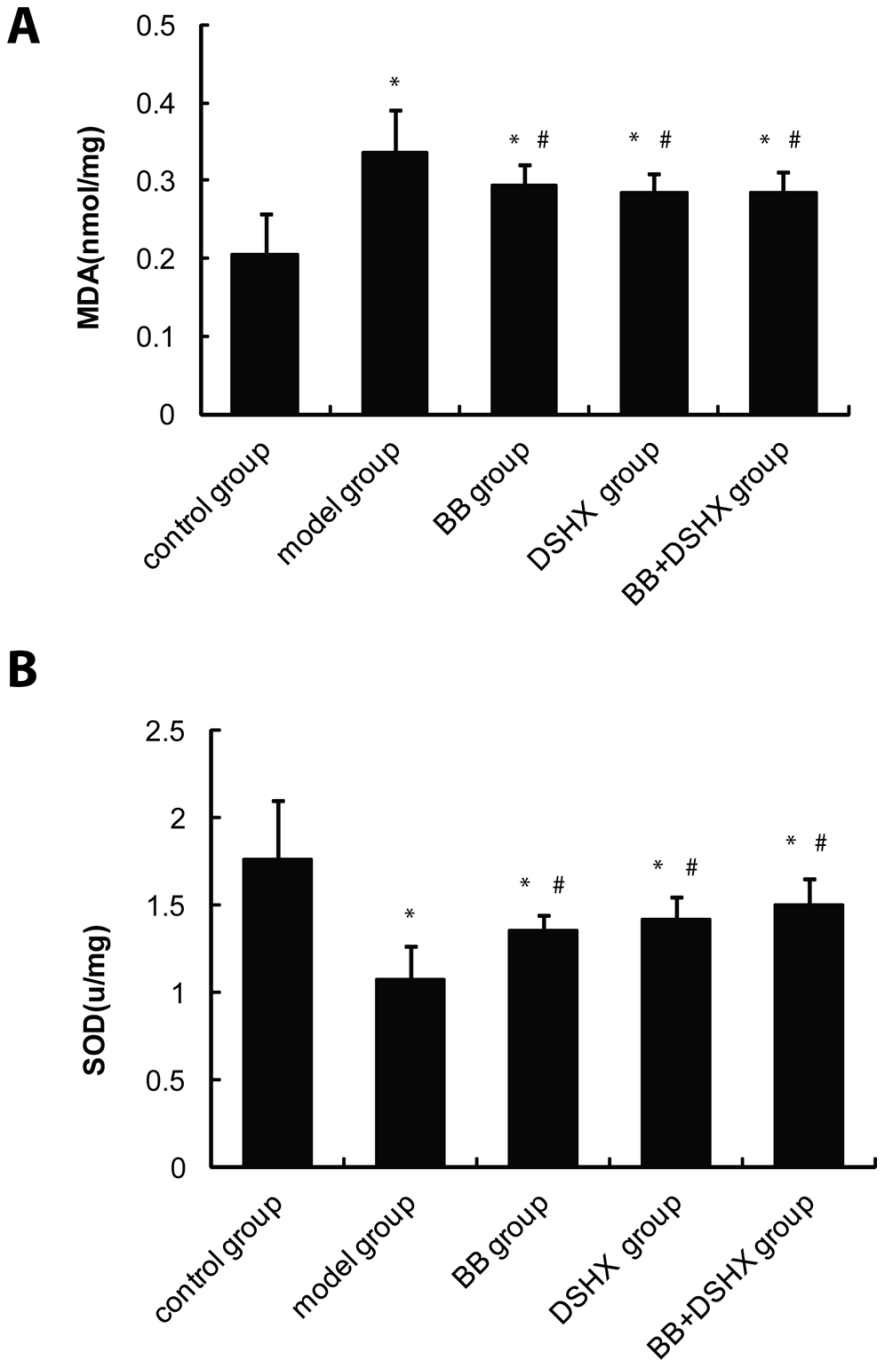
Effect of blueberry juice on hepatic SOD and MDA levels. Liver homogenates were prepared using the frozen liver tissues from rats in the indicated groups. SOD (**A**) and MDA (**B**) levels in the homogenates were measured. *p<0.05 *vs.* the control group, #p<0.05 *vs.* the model group.

Next we wanted to examine whether blueberry juice affected hepatic reactive oxidative species (ROS), which are known to play an important role in CCl_4_-induced liver damage and fibrosis formation. We measured the haptic tissue levels of malondialdehyde (MDA), a marker of oxidative stress. As shown in Figure 4A, blueberry juice consumption significantly reduced MDA levels in the liver compared to those in rats of the fibrosis group that received a control diet. Conversely, the levels of superoxide dismutases (SODs) were significantly increased in the livers of rats receiving blueberry juice feeding compared to rats in the fibrosis group that received a control diet (Figure 4B). These data suggest that the fibrosis protective effect of blueberry juice might be due to its enhancement of SOD activity and thereby decreasing oxidative stress in the liver.

### Blueberry juice upregulates metallothionein (MT) expression in the liver

**Figure 5:**
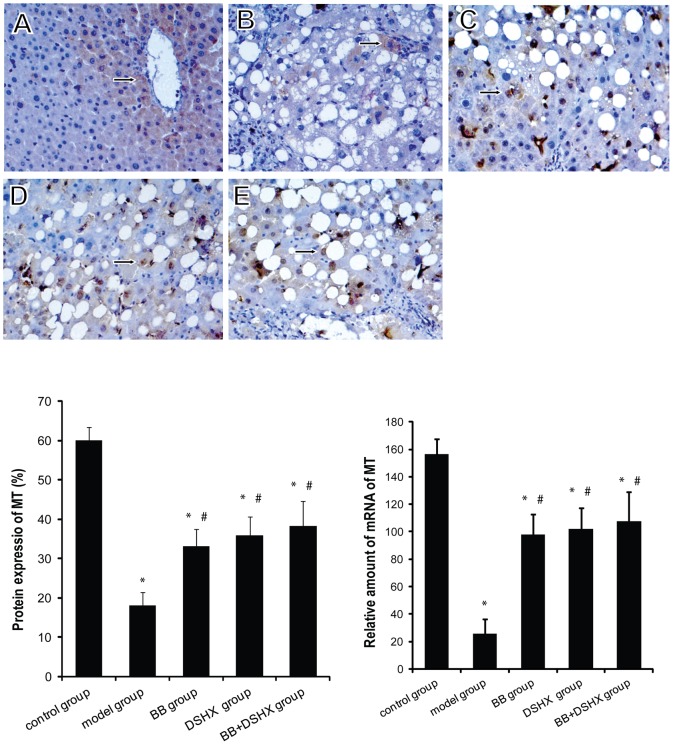
Effect of blueberry juice on MT expression in the rat livers. ***The upper panel images*** show the representative IHC staining of MT protein in the livers of normal control group (**A**), CCl_4_-induced hepatic fibrosis group (the model group, **B**), blueberry juice prevention group (BB group, **C**), Dan-shao-hua-xian capsule prevention group (DSHX group, **D**), and blueberry juice+DSHX prevention group (BB+DSHX group, **E**). Original magnification 400×, arrows indicate positive cytoplasmic/nuclear staining. ***The lower left panel ***shows the quantification data of MT protein expression based on the MT IHC staining. ***The lower right panel*** shows the mRNA levels of MT in the livers assessed by RT-PCR. *p<0.05 *vs.* the control group, #p<0.05 *vs.* the model group.

Metallothionein (MT) is another ROS scavenger with a more efficient antioxidant activity [20], [21],and MT was recently shown to be able to enhance SOD activity in vitro [8]. We performed immunohistochemistry satining and real-time RT-PCR analyses and found that hepatic MT protein and mRNA expression was significantly lower in rats with CCl_4_-induced liver fibrosis; blueberry consumption markedly increased MT expression in the liver (Figure 5).

## Discussion

Our previous work showed that blueberry can increase expression of *Nrf2* and HO-1 in primary hepatic stellate cells [22], and when given orally for 21 days, blueberry can increase the level of mRNA expression of *Nrf2*, Nqo1, and HO-1 in rat liver, and had protective effects against acute and chronic hepatic injury in rats [19], [23]. In the present study, we demonstrated that blueberry juice consumption by rats attenuates CCl_4_-induced hepatic fibrosis. We also showed that blueberry juice can stimulate hepatic expression of metallothionein (MT), increase SOD activity, and reduce oxidative stress in the liver, suggesting that the protection of liver fibrosis by blueberry juice might be through its enhancement of the antioxidative capability of the liver.

Chronic hepatic injury by carbon tetrachloride (CCl_4_) is a well-established animal model of liver fibrosis. Reactive oxygen species and oxidative stress have been shown to play an important role in the etiopathogenesis of the hepatic fibrotic changes [24]–[26], and antioxidant treatment in vivo seems to be effective in preventing or reducing chronic liver damage and fibrosis [27]. Oxidative stress aggravates liver fibrosis via HSC activation, and lipid peroxidation stimulates transcription of the collagen gene. The expression of α-SMA is a characteristic feature of activated HSCs and this is considered as a marker for hepatic fibrosis [28]. Upon liver injury, HSCs become activated and differentiate into myofibroblast-like cells, which proliferate and contribute to collagen deposition in the extracelluar matrix (ECM) [29]. Collagen accounts for about 50% of the total protein in fibrous liver) [30]. Here we showed that blueberry significantly reduces type III collagen content and decrease α-SMA expression in the liver, indicating an inhibitory effect on HSC activation. We also found that blueberry juice can increase SOD activity and decrease oxidative stress in the liver. These data suggest that the fibrosis protective effect of blueberry juice might be due to its antioxidative effect in the liver.

Furthermore, we provided evidence showing that blueberry juice also has a remarkable stimulatory effect on metallothioneins (MTs) expression in the liver. Metallothioneins (MTs) are a family of low molecular mass (6–7 kDa), cysteine-rich, inducible, intracellular proteins that bind heavy metals with high affinity [31]. MT is considered one of the most important intracellular free radical scavengers that plays an important role in defense of stress reactions and tissue injury [32], [33]. It has been shown that mice which have developed a reversible liver fibrosis upon removal of CCl_4_, had a high level of hepatic MT, but mice which developed an irreversible fibrosis, had low expression of MT [34]. Therefore, hepatic MT expression levels are inversely related to the severity of chronic liver damage [35]. Induction of MT synthesis protects animals from hepatotoxicity induced by various toxins including CCl_4_; it also promotes repair and regeneration of injured liver [36]. In this study, we showed that blueberry juice significantly increased MT expression in the liver, which is associated with decreased liver fibrosis, suggesting a potentially important role of MT in blueberry juice-medicated protection against fibrosis.

In summary, our study demonstrated that the liver fibrosis protective effect of blueberry juice was associated with elevated hepatic expression of metallothionein (MT), increased SOD activity, reduced oxidative stress, and decreased levels of α-SMA and Col III in the liver. We therefore propose that dietary supplementation of blueberry juice can augment antioxidative capability of the liver presumably via stimulating MT expression and SOD activity, which in turn promotes HSC inactivation and thereby decreases extracellular matrix collagen accumulation in the liver.
